# Evaluating the knowledge, attitudes, and practices of healthcare workers regarding high‐risk nosocomial infections: A global cross‐sectional study

**DOI:** 10.1002/hsr2.1559

**Published:** 2023-09-11

**Authors:** Elham M. Khatrawi, Priyadarshi Prajjwal, Muhammad Farhan, Pugazhendi Inban, Shraddha Gurha, Saud M. S. Al‐ezzi, Mohammed D. M. Marsool, Prerna Ahuja, Mohammed A. Mateen, Felix O. Aina, Omniat A. Hussin

**Affiliations:** ^1^ Medical Microbiology and Immunology Department Taibah University Medina Saudi Arabia; ^2^ Internal Medicine Bharati Vidyapeeth University Medical College Pune India; ^3^ Internal Medicine Ajman University Ajman UAE; ^4^ Internal Medicine, Government Medical College, Omandurar Chennai Tamil Nadu India; ^5^ Department of Public Health, School of Public Health Poornima University Jaipur India; ^6^ Internal Medicine Lugansk State Medical University Lugansk Ukraine; ^7^ Al‐kindy College of Medicine/University of Baghdad Baghdad Iraq; ^8^ Internal Medicine Teerthankar Mahaveer University Moradabad India; ^9^ Shadan Institute of Medical Sciences Teaching Hospital and Research Centre Hyderabad India; ^10^ Family Medicine, College of Medicine Ekiti State University Ado Ekiti Nigeria; ^11^ Microbiology, Al Manhal Academy Khartoum Sudan

**Keywords:** attitude, healthcare workers, knowledge, nosocomial infections, practice

## Abstract

**Background:**

Healthcare workers (HCWs) play a vital role in delivering care and are frequently exposed to the risk of acquiring infections within the hospital setting. Around 15% of hospitalized patients suffer from these infections globally. However, the role and awareness of HCWs in the transmission of hospital‐acquired infections (HAIs) or nosocomial infections is still unclear. This study aimed to evaluate the knowledge, attitude, and practices (KAP) toward high‐risk microbial infections among HCWs on a global scale to identify measures to address this problem.

**Method:**

A cross‐sectional descriptive study was conducted between 2022 and 2023, with HCWs selected as the study population. Data concerning KAP were collected through a self‐administered online survey questionnaire, using a nonprobability convenience sampling method. Descriptive statistics and regression analysis were used to analyze the data.

**Results:**

A total of 743 HCWs from various countries participated in the study, with the majority of respondents being doctors (64.9%). Data were mainly obtained from Saudi Arabia (26.78%), Iraq (25.84%), India (15.7%), the United States of America (15.2%), and Africa (Sudan, Nigeria) (13.98%). The frequency of good KAP scores among physicians (KAP: 82.5, 80.66, and 70.5), nurses (KAP: 74.1, 73.07, and 88.7), medical practitioners (KAP: 87.2, 77.58, and 75.1), and technicians (KAP: 76.1, 74.38, and 89.6) were obtained as mentioned. With respect to experience, HCWs showed good KAP scores in 1–5 years (KAP: 82.4, 83.3, and 74.1), 5–10 years (KAP: 80.6, 74.54, 83), 10–20 years (KAP: 74.7, 79.1, and 82.7), and >20 years (KAP: 84.6, 78.8, and 82.8) categories.

**Conclusion:**

This study suggests that HCWs have good KAP regarding infection prevention, but there is still room for improvement. Educational seminars and awareness programs can provide better adherence to barrier protection measures such as hand washing, use of gloves, and hand disinfection.

## 1 INTRODUCTION

Hospital‐acquired infections (HAIs) or nosocomial infections are localized or systemic. A healthcare worker (HCW) usually acquires these infections during their interaction with the patients during the examination and other day‐to‐day activities, including the collection of specimens, their processing and discarding, and handling of medical equipment. However, patients acquire these infections during their stay at the hospital.^1^, ^2^ The symptomatology of these infections appears 48–72 h after the patient's admission or within 10 days after the discharge from the healthcare facility.^2^, ^3^, ^4^ People of all age groups are susceptible to these infections, whereas those with weak immune systems, the elderly, and children are the most vulnerable. These infections have been known to severely affect the quality of healthcare services.^2^, ^3^ According to 2011 reports from the US Centers for Disease Control and Prevention (CDC), there were 37,000 HAI‐associated deaths in the US compared to 111,000 in Europe. These infections result in approximately 16 million hospital admissions every year, imposing significant costs and burdens on the healthcare system.^5^ Bacteria such as *Escherichia coli, Staphylococcus aureus, Streptococcus*, and *Clostridium* sp., along with other nonbacterial microorganisms such as viruses, parasites, and fungi, can cause HAIs due to their presence in the air or on various surfaces in healthcare settings. The worldwide estimated rate of these infections is estimated at 12.9% for urinary tract infection (UTI); 21.8% for surgical site infections; 4.0% for lower respiratory tract infections; 21.8% for pneumonia; 9.9% for bloodstream infections; and 5.6% for ENT and mouth infections. Other infections include skin and soft tissues infections accounting for 3.2%, cardiovascular infections 1.2%; bone and joint infections 1.0%; central nervous system (CNS) 0.8%; reproductive tract infections 0.6%; and systemic infections 0.2%.^2^, ^3^, ^6^ The global HCWs' knowledge scores for nosocomial infections remain unknown, despite being the most prevalent causative factor of poor healthcare quality. HAI infections mainly affect low‐ and middle‐income countries (LMICs). Although the overall global incidence of nosocomial infections has reportedly declined over time, the pooled prevalence remains significantly higher in resource‐constrained settings: 15.5% in LMICs compared to 7.6% in high‐income countries. However, this picture of the endemic burden of infections in developing countries is extremely doubtful owing to the scarcity of reliable data.^7^

In recent studies in Pakistan^8^ and Iran,^9^ nurses scored only 43% on knowledge and practice regarding the prevention of HAIs. The lack of knowledge and practice in this area may be a key factor in the spread of these infections.^10^ India reported a 57.5% score,^11^ while two Nigerian tertiary care hospitals orchestrated a 50.8% score for HCWs' practice in the prevention of healthcare‐associated infections.^12^ Two studies from Ethiopia, one conducted in Bahir Dar, revealed a 54.2% practice score of HCWs in the prevention of HAI.^13^ Another study from Amhara regional state referral hospitals in Ethiopia reported 40.7% knowledge scores and 48.7% practice scores for the prevention of nosocomial infections.^14^ Knowledge regarding infectious agents, their mode, and transmission route plays an important role in planning and executing infection control. While inadequate knowledge and incorrect attitudes among HCWs can directly influence their practices and lead to delayed diagnosis, poor infection control practices, and the spread of the disease.^15^ The risk factors for the increased rate of inadequate practice and knowledge toward preventing HAIs include the years of experience, gender distribution among HCWs, their understanding and educational status, and lack of training and adherence to guidelines and workload.^10^

The incidence rate of nosocomial infections in LMIC has been estimated to be very high due to the limited knowledge of professional risks, adherence to universal precautions, and limited availability of personal protective equipment (PPE).^16^ Several epidemiological studies have reported that physicians, nurses, dentists, and laboratory personnel are the main sources of transmission of nosocomial infections due to aseptic precautions during medical procedures. Based on the guidelines of CDC, nosocomial transmission can be efficiently prevented by personal hygiene, use of PPE and proper handling of contaminated beddings and clothing during direct contact with the patients. Another preventive measure could be environmental decontamination which includes utilizing superheated water and sterile patient equipment. Proper training in the prevention of healthcare‐associated infections, incorporated into both preservice and in‐service medical education for healthcare professionals, along with effective monitoring and evaluation methods, can play a crucial role in improving their understanding, awareness, attitude, and practice of universal precautions and infection control when handling patients.^3^ While guidelines are available for HCWs to reduce HAIs,^5^, ^17^ factors such as poor awareness, noncompliance, and logistical and organizational barriers have hindered their effective implementation.^6^ This can be compounded by inadequate knowledge and inappropriate attitudes of HCWs, leading to improper practices that can impact the quality of diagnosis, treatment, and infection control, as well as increase the potential for microbial transmission. Analyzing the knowledge, attitudes, and practices (KAP) of HCWs will assess the transmission risk of nosocomial infections. Additionally, it may help to estimate and compare the quality of treatments in remedial settings. In this regard, the current study envisioned evaluating the attitude, awareness, and practices toward high‐risk microbial infections among HCWs in a global setting to indicate measures to be taken to address the problem.

## 2 METHODOLOGY

A cross‐sectional‐descriptive research study was conducted globally between 2022 and 2023, with HCWs selected as the study population. The study utilized nonprobability sampling to collect responses from a global audience over a specific time interval using a web‐based, and self‐administered survey questionnaire. We distributed the questionnaire through various methods to reach a diverse group of healthcare professionals globally. These methods included online surveys sent to hospitals and healthcare facilities, social media platforms, and personal messaging to individual healthcare professionals. By utilizing these multiple channels, we aimed to capture a wide range of participants from different regions and healthcare settings. Sample size was calculated based on the following factors: Z = standard normal distribution value at 95% confidence level = 1.96, margin of error (*d*) = 5%, and good knowledge = 50%.

### 2.1 Selection criteria

It included the participation of HCWs actively working in hospitals and medical outreach. All HCWs, including doctors, nurses, medical practitioners, technicians (e.g., laboratory technicians, radiologic technologists), pharmacists, midwives, allied health professionals (e.g., physical therapists, occupational therapists, and dieticians), and other hospital staff (e.g., cleaning personnel) were invited to participate in the study. All professionals other than HCWs were excluded from the study.

### 2.2 Definition and classification of HCWs

HCWs and professionals are individuals who work to improve the health of others. They diagnose, treat, and manage diseases, injuries, and health‐related issues based on the needs of the population. The international classification of HCWs includes medical practitioners (generalized and specialists), nursing professionals, midwifery professionals, traditional and complementary medicine professionals, paramedical practitioners, dentists, pharmacists, environmental and occupational health and hygiene professionals, physiotherapists, dieticians and nutritionists, audiologists and speech therapists, optometrists and ophthalmic opticians, medical and pathology laboratory technicians, paramedical technicians and assistants, and medical and dental prosthetic technicians and their assistants.^18^

### 2.3 Design of questionnaire

A comprehensive questionnaire consisting of 27 questions was developed based on guidelines and points from other validated published questionnaires^2^, ^19^ to assess socio‐demographic and sample characteristics, such as age group, profession, years of experience, knowledge, practice, and attitude of HCWs. Names and genders were excluded to maintain participant anonymity. The questionnaire included 12 questions to evaluate knowledge scores, six questions for attitude scores, and five questions to analyze the infection control practices of HCWs. Knowledge was assessed based on the understanding of transmission and sources of infection, hygiene, handling of disposable syringes, those at risk of infection, PPE, and disinfectants that should be used. Situation‐based questions were provided to analyze the attitude of HCWs in several instances. The practice of HCWs was assessed by determining the habits of handwashing, wearing masks, using needles and syringes, and dealing with blood exposure accidents. The level of knowledge, attitudes, and practices was considered appropriate when participants answered 80%–100% of the questions correctly. The questionnaire is available in the Supporting Information: Appendix.

### 2.4 Statistical analysis

Data were statistically analyzed using SPSS (version 23). The normality of the data was determined using the Shapiro‐Wilk test. Descriptive analysis was carried out for qualitative and categorical variables at a nominal scale, which included age, experience, profession, country, KAP variables. The demographic data were presented in frequencies and percentages. The chi‐square test was independently applied to profession and experience to determine the association with KAP variables. Cramer's *v* was used to assess the correlation between profession and experience with knowledge, practice, and attitudes. A range of 0–1 was used to depict the strength of the correlation between the two variables, where 0 indicated a weak correlation while 1 indicated a strong correlation. Binary logistic regression with Hosmer Lemeshow goodness of fit was applied to determine the relationship between the understanding of HCWs with the route of transmission of infection and their practice. The results were depicted as odds ratio (OR), where odds of >1 indicated a high rate of practice while odds <1 indicated decreased practice, with 95% confidence intervals (CIs). A *p*‐value of <0.05 was considered an indicator of statistical significance. The statistical tests were two‐sided.

### 2.5 Consent for publication

Before data collection, all study participants signed an informed consent form that addressed the purpose, significance, and privacy concerns of the study.

### 2.6 Ethical approval

The study obtained ethical approval from the Taibah University College of Medicine Research Ethics Committee (Study ID/Ref no.: TU‐019‐22) and Ajman University Research Ethics Committee (Ref no.: MFS6Feb) on February 15th and February 6th respectively. Before data collection, all study participants signed an informed consent form that addressed the purpose, significance, and privacy concerns of the study. Confidentiality and anonymity of the participants' data were assured, and they had the right to refuse or withdraw from the study at any point in time. No incentives or rewards were offered to the participants.

## 3 RESULTS

**Figure 1:**
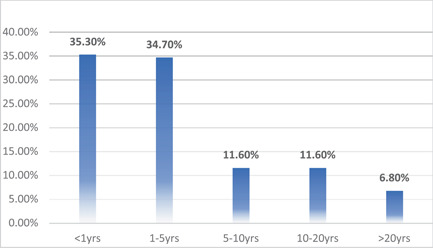
Frequency of healthcare workers (HCWs) work experience.

**Figure 2:**
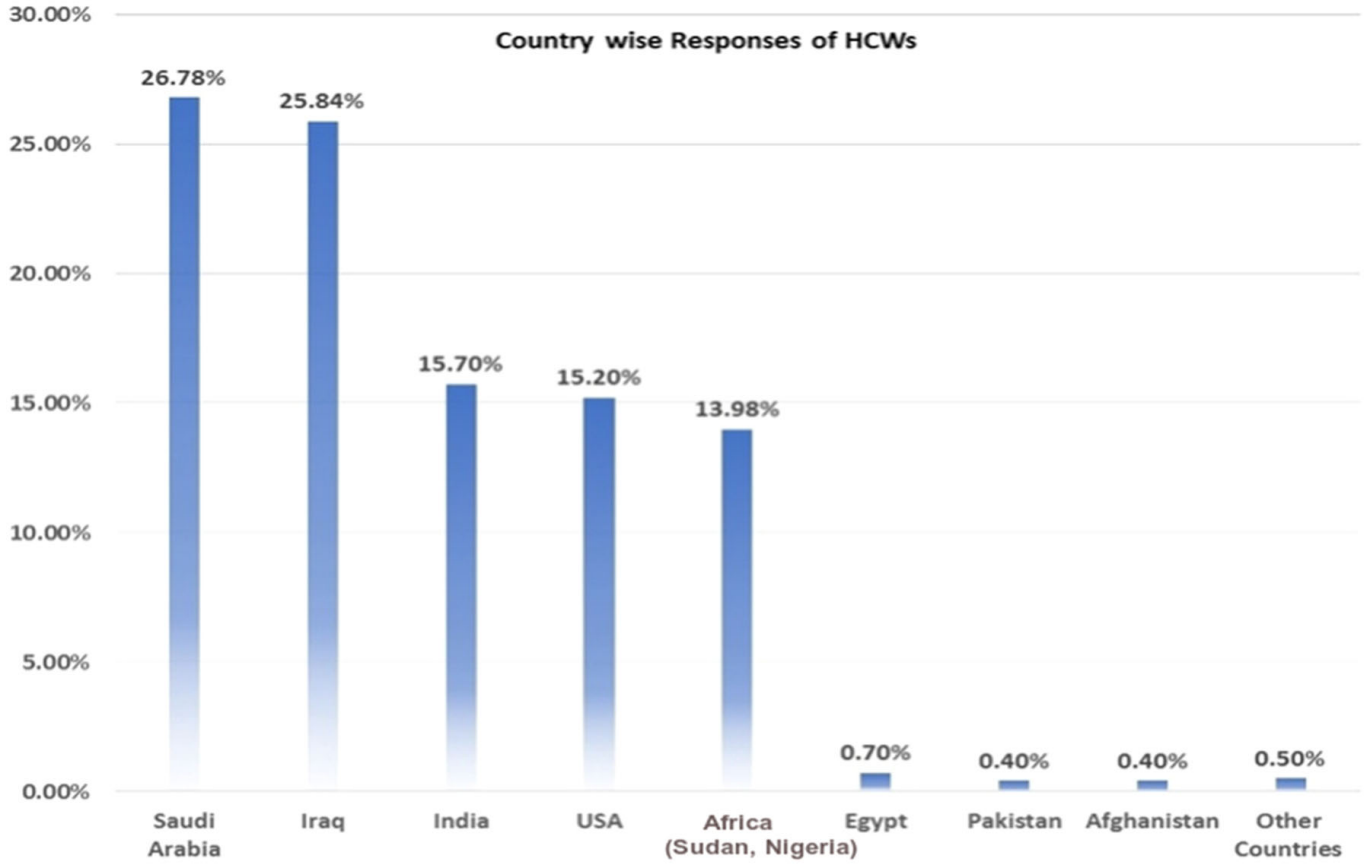
Country wise responses of healthcare workers (HCWs).

**Figure 3:**
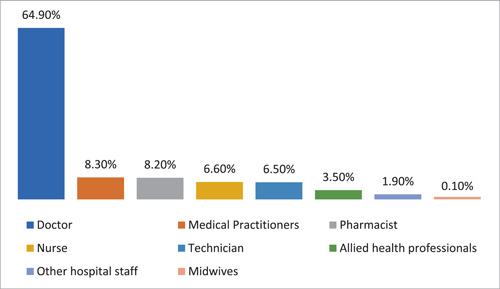
Professional distribution of healthcare workers (HCWs).

**Figure 4:**
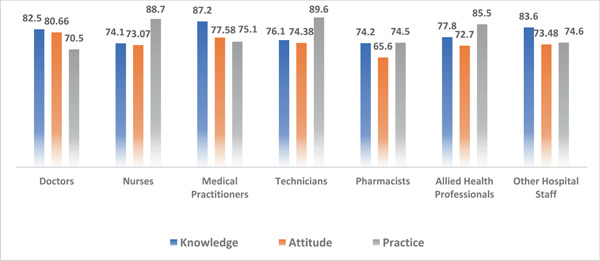
Knowledge, attitude, and practices (KAP) scores based on the profession of healthcare workers (HCWs).

**Figure 5:**
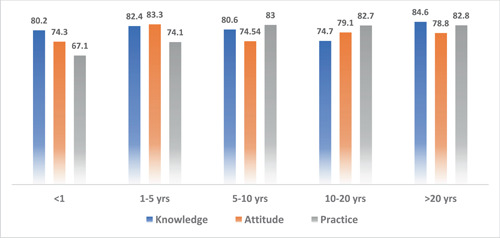
Knowledge, attitude, and practices (KAP) scores based on the experience of healthcare workers (HCWs).

A total of 743 respondents participated in the study. Among them, 404 (4.4%) respondents were in 17–25 age bracket, 210 (28.3%) in 25–35, 73 (9.8%) in 35–45, and 56 (7.5%) in the 45–60 age groups. Other sociodemographic characteristics that were taken into consideration included years of experience (Figure 1), healthcare profession (Figure 2), and location/country (Figure 3). About 35.3% of the study participants had less than 1 year of experience, while 34.7% had between 1 and 5 years of experience (Figure 1). The majority of respondents (64.9%) were doctors, as shown in (Figure 3). We primarily obtained data from Saudi Arabia (26.78%), Iraq (25.84%), India (15.7%), the United States (15.2%), Africa (13.98%), Egypt, (0.7%), UAE (0.7%), Pakistan (0.4%), and Afghanistan (0.4%). Other countries accounted for 0.5% including Bangladesh (0.1%), Oman (0.1%), Qatar (0.1%), Russia (0.1%), and Canada (0.1%) (Figure 2). Figure 4 depicts the frequency of good KAP scores among physicians (KAP: 82.5, 80.66, and 70.5), nurses (KAP: 74.1, 73.07, and 88.7), medical practitioners (KAP: 87.2, 77.58, and 75.1), and technicians (KAP: 76.1, 74.38, and 89.6) (Table 1). With regard to experience (Figure 5), HCWs highlighted good KAP scores in 1–5 years (KAP: 82.4, 83.3, and 74.1), 5–10 years (KAP: 80.6, 74.54, and 83), 10–20 years (KAP: 74.7, 79.1, and 82.7), and >20 years (KAP: 84.6, 78.8, and 82.8) categories (Table 1, Figure 5).

**Table 1:** Knowledge, attitude, and practices (KAP) scores based on the profession and experience of healthcare workers (HCWs).

	Knowledge	Attitude	Practice	Total
Profession				
Doctors	82.5	80.66	70.5	77.9
Nurses	74.1	73.07	88.7	78.6
Medical practitioners	87.2	77.58	75.1	80.0
Technicians	76.1	74.38	89.6	80.0
Pharmacists	74.2	65.6	74.5	71.4
Allied health professionals	77.8	72.7	85.5	78.7
Other hospital staff	83.6	73.48	74.6	77.2
Experience (years)				
<1	80.2	74.3	67.1	73.9
1–5	82.4	83.3	74.1	79.9
5–10	80.6	74.54	83	79.4
10–20	74.7	79.1	82.7	78.8
>20	84.6	78.8	82.8	82.1

There was a significantly favorable professional attitude of HCWs toward HAI prevention. They strongly rejected the reuse of catheters and surgical forceps, stressed the importance of glove use during patient contact, and acknowledged the high HAI risk (Table 2). The study also demonstrated a strong correlation between HCWs' experience levels and their attitudes (Table 3).

**Table 2:** Correlation of healthcare workers' (HCWs) attitude and profession.

Attitude	Profession (%)									
	Doctors	Nurses	Medical professional	Technicians	Pharmacists	Midwives	Allied health professionals	Other hospital staff	*p*‐Value	Cramer's *v*
**List of reusable equipment**										
Syringes										
Yes	45 (9.3)	6 (12.2)	10 (16.1)	6 (12.5)	11 (18.0)	0 (0)	4 (15.4)	1 (7.1)	0.426	0.097
No	437 (90.7)	43 (87.8)	52 (83.9)	42 (87.5)	50 (82.0)	1 (100)	22 (84.6)	13 (92.9)		
Stethoscope										
Yes	424 (88.0)	44 (89.8)	53 (85.5)	44 (91.7)	45 (73.8)	1 (100)	22 (84.6)	12 (84.7)	0.124	0.124
No	58 (12.0)	5 (10.2)	9 (14.5)	4 (8.3)	16 (26.2)	0 (0)	4 (15.4)	2 (14.3)		
Catheters										
Yes	81 (16.8)	29 (59.2)	14 (22.6)	24 (50.0)	19 (31.1)	0 (0)	13 (50.0)	3 (21.4)	<0.001*	0.318
No	401 (83.2)	20 (40.8)	48 (77.4)	24 (50.0)	42 (68.9)	1(100)	13 (50.0)	11 (78.6)		
Endoscope										
Yes	346 (71.8)	34 (69.4)	44 (71.0)	42 (87.5)	42 (68.9)	0(0)	15 (57.7)	12 (85.7)	0.078	0.131
No	136 (28.2)	15 (30.6)	18 (29.0)	6 (12.5)	19 (31.1)	1 (100)	11 (42.3)	2 (14.3)		
Surgical sponges										
Yes	50 (10.4)	5 (10.2)	7 (11.3)	2 (4.2)	5 (8.2)	0 (0)	3 (11.5)	3 (21.4)	0.733	0.077
No	432 (89.6)	44 (89.8)	55 (88.7)	46 (95.8)	56 (91.8)	1 (100)	23 (88.5)	11 (78.6)		
Surgical forceps										
Yes	333 (69.1)	17 (34.7)	40 (64.5)	17 (35.4)	20 (32.8)	1 (100)	13 (50.0)	5 (35.7)	<0.001*	0.299
No	149 (30.9)	32 (65.3)	22 (35.5)	31 (64.6)	41 (67.2)	0 (0)	13 (50.0)	9 (64.3)		
**Average percentage of HCWs risk of developing hospital‐acquired infections (HAIs) (between 0% and 100%)**										
0%–25%	167 (34.6)	7 (14.3)	6 (9.7)	5 (10.4)	9 (14.8)	0 (0)	2 (7.7)	1 (7.1)	<0.001*	0.185
25%–50%	98 (20.3)	3 (6.1)	14 (22.6)	5 (10.4)	9 (14.8)	1 (100)	3 (11.5)	3 (21.4)		
50%–75%	75 (15.6)	8 (16.3)	13 (21.0)	9 (18.8)	10 (16.4)	0 (0)	7 (26.9)	3 (21.4)		
75%–100%	37 (7.7)	11 (22.4)	8 (12.9)	14 (29.2)	8 (13.1)	0 (0)	8 (30.8)	0 (0)		
I do not know	105 (21.8)	20 (40.8)	21 (33.9)	15 (31.3)	25 (41.0)	0 (0)	6 (23.1)	7 (50.0)		
**Wearing similar gloves while contacting different patients**										
True	72 (14.9)	1 (2.0)	14 (22.6)	7 (14.6)	13 (21.3)	0 (0)	5 (19.2)	2 (14.3)	0.121	0.124
False	410 (85.1)	48 (98.0)	48 (77.4)	41 (85.4)	48 (78.7)	1 (100)	21 (80.8)	12 (85.7)		
**Wearing gloves while having a direct contact when a patient seems healthy**										
Yes	404 (83.8)	44 (89.8)	46 (74.2)	38 (79.2)	37 (60.7)	0 (0)	19 (73.1)	10 (71.4)	<0.001*	0.197
No	78 (16.2)	5 (10.2)	16 (25.8)	10 (20.8)	24 (39.3)	1 (100)	7 (26.9)	4 (28.6)		
**Best way of dealing with a hazard to ensure others are not put at risk**										
Remove it immediately	312 (64.7)	27 (55.1)	40 (64.5)	26 (54.2)	256 (42.6)	1 (00)	19 (73.1)	6 (42.9)	<0.001*	0.18
Leave it for others to sort out	27 (5.6)	9 (18.4)	3 (4.8)	12 (25.0)	2 (3.3)	0 (0)	3 (11.5)	2 (14.3)		
Place a barrier tape around it	54 (11.2)	7 (14.3)	11 (17.7)	7 (14.6)	21 (34.4)	0 (0)	1 (3.8)	1 (7.1)		
Display a notice or warning sign	89 (18.5)	6 (12.2)	8 (12.9)	3 (6.3)	12 (19.7)	0 (0)	3 (11.5)	5 (35.7)		
**Using goggles while cutting catheter bags, cleaning bedpans, and emptying suction cups**										
Yes	429 (89.0)	37 (75.5)	55 (88.7)	37 (77.1)	52 (85.2)	1 (100)	22 (84.6)	11 (78.6)	0.078	0.131
No	53 (11.0)	12 (24.5)	7 (11.3)	11 (22.9)	9 (14.8)	0 (0)	4 (15.4)	3 (21.4)		

* Statistically significant at *p* < 0.05.

**Table 3:** Correlation of healthcare workers' (HCWs) attitude and experience.

Attitude	Years of experience (%)						
	<1	01‐May	05‐October	20‐October	>20	*p*‐Value	Cramer's *v*
**List of reusable equipment**							
Syringes							
Yes	39 (14.9)	22 (8.5)	11 (12.8)	9 (10.5)	2 (3.9)	0.077	0.107
No	223 (85.1)	236 (91.5)	75 (87.2)	77 (89.5)	49 (96.1)		
Stethoscope							
Yes	223 (85.1)	225 (87.2)	73 (84.9)	77 (84.9)	47 (92.2)	0.591	0.061
No	39 (4.9)	33 (12.8)	13 (15.1)	13 (15.1)	4 (7.8)		
Catheters							
Yes	48 (18.3)	48 (18.3)	34 (39.5)	33 (38.4)	20 (39.2)	<0.001*	0.219
No	214 (81.7)	210 (81.4)	52 (60.5)	53 (61.6)	31 (60.8)		
Endoscope							
Yes	153 (58.4)	202 (78.3)	65 (75.6)	75 (87.2)	40 (78.4)	<0.001*	0.234
No	109 (41.6)	56 (21.7)	21 (24.4)	11 (12.8)	11 (21.6)		
Surgical sponges							
Yes	38 (14.5)	25 (9.7)	5 (5.8)	5 (5.8)	2 (3.9)	0.024*	0.123
No	224 (85.5)	233 (90.3)	81 (94.2)	81 (94.2)	49 (96.1)		
Surgical forceps							
Yes	157 (59.9)	171 (66.3)	36 (41.9)	51 (59.3)	31 (60.8)	0.003*	0.147
No	105 (40.1)	87 (33.7)	50 (58.1)	35 (40.7)	20 (39.2)		
**Average percentage of HCWs at risk of developing healthcare‐associated infection (between 0% and 100%)**							
0%–25%	61 (23.3)	103 (39.9)	7 (8.1)	14 (16.3)	12 (23.5)	<0.001*	0.192
25%–50%	65 (24.8)	46 (17.8)	14 (16.3)	7 (8.1)	4 (7.8)		
50%–75%	53 (20.2)	38 (14.7)	13 (15.1)	15 (7.4)	6 (11.8)		
75%–100%	16 (6.1)	14 (5.4)	19 (22.1)	24 (27.9)	13 (25.5)		
I do not know	25.6	57 (22.1)	33 (38.4)	26 (30.2)	16 (31.4)		
**Wearing similar gloves while contacting different patients**							
True	48 (18.3)	39 (15.1)	12 (14.0)	10 (11.6)	5 (9.8)	0.4	0.074
False	214 (81.7)	219 (84.9)	74 (86.0)	76 (88.4)	46 (90.2)		
**Wearing gloves while having a direct contact when a patient seems healthy**							
Yes	207 (79.0)	217 (84.1)	67 (77.9)	71 (82.6)	36 (70.6)	0.178	0.092
No	55 (21.0)	41 (15.9)	19 (22.1)	15 (17.4)	15 (29.4)		
**Best way of dealing with a hazard to ensure others are not put at risk**							
Remove it immediately	137 (52.3)	189 (73.3)	45 (52.3)	50 (58.1)	36 (70.6)	<0.001*	0.149
Leave it for others to sort out	19 (7.3)	12 (4.7)	10 (11.6)	15 (17.4)	2 (3.9)		
Place a barrier tape around it	40 (15.3)	30 (11.6)	17 (19.8)	9 (10.5)	6 (11.8)		
Display a notice or warning sign	66 (25.2)	27 (10.5)	14 (16.3)	12 (14.0)	7 (13.7)		
**Using goggles while cutting catheter bags, cleaning bedpans, and emptying suction cups**							
Yes	221 (84.4)	240 (93.0)	73 (84.9)	73 (84.9)	37 (72.5)	0.001*	0.162
No	41 (15.6)	18 (7.0)	13 (15.1)	13 (15.1)	14 (27.5)		

* Statistically significant at *p* < 0.05.

### 3.1 Knowledge of HCWs about HAI prevention

#### 3.1.1 Correlation of HCWs' professional nature and knowledge scores

The results of the study showed significant variations in the knowledge scores of HCWs based on their profession, particularly regarding the transmission of infections through blood‐borne viruses like Hepatitis C and D (*p* < 0.002), chickenpox/shingles (*p* < 0.001), tuberculosis (*p* < 0.01), and HIV (*p* < 0.001). We additionally observed significant differences in responses related to the transmissibility of skin and soft tissue infections through routes such as kissing (*p* < 0.001), sexual intercourse (*p* < 0.003), and respiratory transmission (*p* < 0.001). The study further identified significant variations (*p* < 0.001) in the knowledge of HCWs through respiratory transmission. The response on transmission modes of diarrheal illnesses showed fluctuations considering respiratory (*p* < 0.007) and fecal‐oral routes (*p* < 0.001). The analysis of HCWs' technical knowledge revealed an uneven distribution with respect to hygienic practices, such as the reuse of syringes (*p* < 0.015) and preferences for cleaning methods (*p* < 0.001) (Table 4).

**Table 4:** Correlation of healthcare workers' (HCWs) knowledge and profession.

Knowledge	Profession (%)									
	Doctors	Nurses	Medical professional	Technicians	Pharmacists	Midwives	Allied health professionals	Other hospital staff	*p*‐Value	Cramer's *v*
**Microbes that are mainly transmitted through blood**										
Influenza										
Yes	34 (7.1)	3 (6.1)	6 (9.7)	0 (0)	6 (9.8)	0 (0)	2 (7.7)	3 (21.4)	0.237	0.112
No	448 (92.9)	46 (93.9)	56 (90.3)	48 (100)	55 (90.2)	1 (100)	24 (92.3)	11 (78.6)		
Hepatitis C and D virus										
Yes	285 (59.1)	45 (91.8)	44 (71.0)	47 (98.9)	46 (75.4)	1 (100)	23 (88.5)	11	<0.001*	0.278
								−78.6		
No	197 (40.9)	4 (8.2)	18 (29.0)	1 (2.1)	15 (24.6)	0 (0)	3 (11.5)	3 (21.4)		
Tuberculosis										
Yes	50 (10.4)	5 (10.2)	8 (12.9)	4 (8.3)	18 (29.5)	0 (0)	4 (51.4)	5 (35.7)	<0.01*	0.187
No	432 (89.6)	44 (89.8)	54 (87.1)	44 (91.7)	43 (70.5)	1 (100)	22 (84.6)	9 (64.3)		
Chicken pox and Shingles (caused by herpes virus)										
Yes	34 (7.1)	6 (12.2)	5 (8.1)	5 (8.1)	15 (24.6)	0 (0)	8 (30.8)	4 (28.6)	<0.001*	0.221
No	448 (92.9)	43 (87.8)	57 (91.9)	43 (89.6)	46 (75.4)	1 (100)	18 (69.2)	10 (71.4)		
HIV										
Yes	165 (34.2)	0 (0)	12 (19.4)	0 (0)	7 (11.5)	0 (0)	1 (3.8)	1 (7.1)	<0.001*	0.307
No	317 (65.8)	49 (100)	50 (80.6)	48 (100)	54 (88.5)	1 (100)	25 (96.2)	13 (92.9)		
70% alcohol kills germs										
Yes	401 (83.2)	43 (87.8)	51 (82.3)	47 (97.9)	54 (88.5)	1 (100)	25 (96.2)	10 (71.4)	0.06	0.135
No	81 (16.8)	6 (12.2)	11 (17.7)	1 (2.1)	7 (11.5)	0 (0)	1 (3.8)	4 (28.6)		
Healthy person is capable of transmitting infections										
Yes	458 (95)	43 (87.8)	58 (93.5)	41 (85.4)	52 (85.2)	1 (100)	20 (76.9)	11 (78.6)	0.001*	0.186
No	24 (5.0)	6 (12.2)	4 (6.5)	7 (14.6)	9 (14.8)	0 (0)	6 (23.1)	3 (21.4)		
Sharing of food with patients spreads viral or bacterial infections										
Yes	394 (81.7)	38 (77.6)	53 (85.5)	41 (85.4)	44 (72.1)	1 (100)	18 (69.2)	12 (85.7)	0.348	0.103
No	88 (18.3)	11 (22.4)	9 (14.5)	7 (14.6)	17 (27.9)	0 (0)	8 (30.8)	2 (14.3)		
Cleaning hands while caring for patients with vomiting or diarrheal illnesses										
Alcohol	89 (18.5)	15 (30.6)	19 (30.6)	14 (29.2)	21 (34.4)	0 (0)	8 (30.8)	4 (28.6)	<0.001*	0.221
Soap	177 (36.7)	5 (10.2)	9 (14.5)	1 (2.1)	7 (11.5)	0 (0)	0 (0)	2 (14.3)		
Any of them	216 (44.8)	29 (59.2)	34 (54.8)	33 (68.8)	33 (54.1)	1 (100)	18 (69.2)	8 (57.1)		
**Bending, shearing, breaking, or re‐capping of disposable needles or removal from disposable syringes**										
Yes	116 (24.1)	5 (10.2)	18 (29.0)	2 (4.2)	12 (19.7)	0 (0)	4 (15.4)	4 (28.6)	0.015*	0.153
No	366 (75.9)	44 (89.8)	44 (71.0)	46 (95.8)	49 (80.3)	1 (100)	22 (84.6)	10 (71.4)		
**Can we empty the contents of the sharp's disposal container into another container?**										
Yes	80 (16.6)	7 (14.3)	16 (25.8)	8 (16.7)	16 (26.2)	45 (73.8)	9 (34.6)	5 (35.7)	0.06	0.135
No	402 (83.4)	42 (85.7)	46 (74.2)	40 (83.3)	0 (0)	1 (100)	17 (65.4)	9 (64.3)		
**Only elderly, comorbid patients, and people with weak immune systems are affected by hospital‐acquired infections (HAIs)**										
Yes	112 (23.2)	8 (16.3)	17 (27.4)	7 (14.6)	18 (29.5)	0 (0)	4 (15.4)	4 (28.6)	0.458	0.095
No	370 (76.8)	41 (83.7)	45 (72.6)	41 (85.4)	43 (70.5)	1 (100)	22 (84.6)	10 (71.4)		
**Wearing medical gowns during surgical procedures only**										
True	123 (25.5)	8 (16.3)	12 (19.4)	8 (16.7)	14 (23.0)	0 (0)	8 (30.8)	5 (35.7)	0.493	0.093
False	359 (74.5)	4 (83.7)	50 (80.6)	40 (83.3)	47 (77.0)	1 (100)	18 (69.2)	9 (64.3)		
**Transmission of skin and soft tissue infections**										
Skin contact										
Yes	450 (93.4)	48 (98.0)	56 (90.3)	44 (91.7)	57 (93.4)	1 (100)	23 (88.5)	13 (92.9)	0.811	0.071
No	32 (6.6)	1 (2.0)	6 (9.7)	4 (8.3)	−6.6	0 (0)	3 (1.0)	1 (7.1)		
Sexual intercourse										
Yes	241 (50)	31 (63.3)	43 (69.4)	32 (66.7)	32 (52.5)	1 (100)	11 (42.3)	12 (85.7)	0.003*	0.171
No	241 (50)	18 (36.7)	19 (30.6)	16 (33.3)	29 (47.5)	0 (0)	15 (57.7)	2 (14.)		
Respiratory transmission										
Yes	95 (19.7)	4 (8.2)	17 (27.4)	1 (2.1)	10 (16.5)	0 (0)	1 (3.8)	6 (42.9)	0.001*	0.186
No	387 (80.3)	45 (91.8)	45 (72.6)	47 (97.9)	51 (83.6)	1 (100)	25 (96.2)	8 (57.1)		
Fecal‐oral route transmission										
Yes	50 (10.4)	3 (6.1)	4 (6.5)	4 (8.3)	8 (13.1)	0 (0)	3 (11.5)	1 (7.1)	0.895	0.062
No	432 (89.6)	46 (93.9)	58 (93.5)	44 (91.7)	53 (86.9)	1 (100)	23 (55.5)	13 (92.9)		
Kissing										
Yes	328 (68.0)	23 (46.9)	40 (64.5)	21 (43.8)	25 (41.0)	1 (100)	10 (38.5)	8 (57.1)	<0.001*	0.223
No	154 (32.0)	26 (53.1)	22 (35.5)	27 (56.3)	36 (59.0)	0 (0)	16 (61.5)	6 (42.9)		
**Transmission of respiratory infections**										
Air droplets										
Yes	443 (91.9)	47 (95.9)	57 (91.9)	44 (91.7)	52 (85.2)	1 (100)	24 (92.3)	13 (92.9)	0.706	0.079
No	39 (8.1)	2 (4.1)	5 (8.1)	4 (8.1)	9 (14.8)	0 (0)	2 (7.7)	1 (7.1)		
Fecal oral route										
Yes	67 (13.9)	4 (8.2)	8 (12.9)	1 (2.1)	9 (14.8)	0 (0)	1 (3.8)	1 (7.1)	0.241	0.111
No	415 (86.1)	45 (91.8)	54 (87.1)	47 (97.9)	52 (85.2)	1 (100)	25 (96.2)	13 (92.9)		
Respiratory secretions										
Yes	421 (87.3)	47 (95.9)	52 (83.9)	47 (97.9)	52 (85.2)	1 (100)	24 (92.3)	13 (92.9)	0.183	0.117
No	61 (12.7)	2 (4.1)	10 (16.1)	1 (2.1)	9 (14.8)	0 (0)	2 (7.7)	1 (7.1)		
Sexual intercourse										
Yes	38 (7.9)	3 (6.1)	6 (9.7)	5 (10.4)	9 (14.8)	0 (0)	2 (7.7)	3 (21.4)	0.46	0.095
No	444 (92.1)	46 (93.9)	56 (90.3)	43 (89.6)	52 (85.2)	1 (100)	24 (92.3)	11 (78.6)		
Contact with contaminated hands										
Yes	163 (33.8)	3(6.1)	17(27.4)	2(4.2)	18(29.5)	1(100)	1(3.8)	9 (64.3)	<0.001*	0.263
No	319 (66.2)	46(93.9)	45(72.6)	46(95.8)	43(70.5)	0(0)	25(96.2)	5 (35.7)		
**Spread of diarrheal illnesses**										
Air Droplets										
Yes	54 (11.2)	2 (4.1)	5 (8.1)	5 (10.4)	13 (21.3)	1 (100)	3 (11.5)	2 (14.3)	0.017*	0.152
No	428 (88.8)	47 (95.9)	57 (91.9)	43 (89.6)	48 (78.7)	0 (0)	23 (88.5)	12 (85.7)		
Fecal oral route										
Yes	416 (83.3)	41 (83.7)	48 (77.4)	38 (79.2)	38 (62.3)	1 (100)	18 (69.2)	10 (71.4)	<0.001*	0.193
No	66 (13.7)	8 (16.3)	14 (22.6)	10 (20.8)	23 (37.7)	0	8 (30.8)	4 (28.6)		
Respiratory secretions										
Yes	37 (7.7)	1 (2.0)	4 (6.5)	5 (10.4)	9 (14.8)	1 (100)	4 (15.4)	1 (7.1)	0.007*	0.161
No	445 (92.3)	48 (98.0)	58 (93.5)	43 (89.6)	52 (85.2)	0 (0)	22 (84.6)	13 (92.9)		
Sexual intercourse										
Yes	56 (11.6)	3 (6.1)	7 (11.3)	9 (18.8)	13 (21.3)	0 (0)	0 (0)	3 (21.4)	0.064	0.134
No	426 (88.4)	46 (93.9)	55 (88.7)	39 (81.3)	48 (78.7)	1 (100)	26 (100)	11 (78.6)		
Contact with contaminated hands										
Yes	394 (81.7)	40 (81.6)	50 (80.6)	37 (77.1)	41 (67.2)	1 (100)	19 (73.1)	12 (85.7)	0.268	0.109
No	88 (18.3)	9 (18.2)	12 (19.2)	11 (22.9)	20 (32.8)	0 (0)	7 (26.9)	2 (14.3)		

* Statistically significant at *p* < 0.05.

#### 3.1.2 Correlation of HCWs' experience and knowledge scores

In relation to their experience, HCWs exhibited significantly higher levels of knowledge with respect to the bloodborne dissemination of infectious organisms, such as Hepatitis C and D (*p* < 0.001) and HIV (*p* < 0.001). Moreover, their responses to the transmission of soft tissue infections through sexual intercourse (*p* < 0.002), respiratory route (*p* < 0.001), or kissing (*p* < 0.001) also displayed significant differences. Experienced HCWs acknowledged the importance of safeguarding vulnerable populations, such as the elderly and immunocompromised individuals, against HAIs, and the use of PPE (*p* < 0.002). Similarly, the general understanding of the fecal‐oral route as a source of diarrheal infections was markedly high (*p* < 0.004) among experienced HCWs. Furthermore, their technical knowledge regarding hygiene practices, including the use of 70% alcohol (*p* < 0.001), proper disposal of syringes (*p* < 0.001), and cleaning methods preferences (*p* < 0.001) also varied significantly (Table 5).

**Table 5:** Correlation of healthcare workers' (HCWs) knowledge and experience.

Knowledge	Years of experience (%)						
	<1	1–5	5–10	10–20	>20	*p*‐Value	Cramer's *v*
**Microbes that are mainly transmitted through blood**							
Influenza							
Yes	25 (9.5)	20 (7.8)	6 (7.0)	2 (2.3)	1 (2.0)	0.118	0.100
No	237 (90.5)	238 (92.2)	80 (93.0)	84 (97.7)	50 (98.0)		
Hepatitis C and D virus							
Yes	180 (68.7)	131 (50.8)	75 (87.2)	72 (83.7)	44 (86.3)	<0.001*	0.300
No	82 (31.3)	127 (49.2)	11 (12.8)	14 (6.3)	7 (13.7)		
Tuberculosis causing bacterium							
Yes	39 (14.9)	32 (12.4)	11 (12.8)	8 (9.3)	4 (7.8)	0.535	0.065
No	223 (85.1)	226 (87.6)	75 (87.2)	78 (90.7)	47 (92.2)		
Chicken pox and Shingles (caused by Herpes Virus)							
Yes	31 (11.8)	22 (8.5)	8 (9.3)	8 (9.3)	8 (15.7)	0.508	0.067
No	231 (88.2)	236 (91.5)	78 (90.7)	78 (90.7)	43 (84.3)		
HIV							
Yes	60 (22.9)	108 (41.9)	3 (3.5)	8 9.3)	7 (13.7)	<0.001*	0.319
No	202 (77.1)	150 (58.1)	83 (96.5)	78 (90.7)	44 (86.3)		
**70% alcohol kills germs**							
Yes	201 (76.7)	225 (87.5)	77 (89.5)	81 (94.2)	48 (94.1)	<0.001*	0.186
No	61 (23.3)	33 (412.8)	9 (10.5)	5 (5(5.8)	3 (5.9)		
**Healthy person is capable of transmitting infections**							
Yes	245 (93.5)	237 (91.9)	76 (88.4)	78 (90.7)	48 (94.1)	0.578	0.062
No	17 (6.5)	21 (8.1)	10 (11.6)	8 (9.3)	3 (5.9)		
**Sharing of food with patients spreads viral or bacterial infections**							
Yes	208 (79.4)	224 (86.8)	66 (76.7)		37 (72.5)	0.033*	0.119
No	54 (20.6)	34 (13.2)	20 (23.3)		14 (27.5)		
**Cleaning hands while caring for patients with vomiting or diarrheal illnesses**							
Alcohol	84 (32.1)	34 (13.2)	21 (24.4)	24 (27.9)	7 (13.7)	<0.001*	0.226
Soap	72 (27.5)	101 (39.1)	7 (8.1)	6 (7.0)	15 (29.4)		
Any of them	106 (40.5)	123 (47.7)	58 (67.4)	56 (65.1)	29 (56.9)		
**Bending, shearing, breaking, or re‐capping of disposable needles or removal from disposable syringes**							
Yes	79 (30.2)	45 (17.4)	17 (19.8)	13 (15.1)	7 (13.7)	0.001*	0.156
No	183 (69.8)	213 (82.6)	69 (80.2)	73 (84.9)	44 (86.3)		
**Can we empty the contents of the sharp's disposal container into another container?**							
Yes	54 (20.6)	39 (15.1)	21 (24.4)	19 (22.1)	8 (15.7)	0.238	0.086
No	208 (79.4)	219 (84.9)	65 (75.6)	67 (77.9)	43 (84.3)		
**Only elderly, comorbid patients, and people with weak immune system are affected by hospital acquired illnesses**							
Yes	80 (30.5)	51 (9.8)	19 (22.1)	11 (12.8)	9 (17.6)	0.003*	0.146
No	182 (68.5)	207 (80.2)	67 (779)	75 (87.2)	42 (82.4)		
**Wearing medical gowns during surgical procedures only**							
True	80 (30.5)	63 (24.4)	10 (11.6)	13 (15.1)	12 (23.5)	0.002*	0.152
False	182 (69.5)	195 (75.6)	76 (88.4)	73 (84.9)	39 (76.5)		
**Transmission of skin and soft tissue infections**							
Skin contact							
Yes	239 (91.2)	241 (93.4)	80 (93.0)	81 (94.2)	51 (100)	0.245	0.086
No	23 (8.8)	17 (6.6)	6 (7.0)	5 (5.8)	0 (0)		
Sexual intercourse							
Yes	153 (58.4)	114 (44.2)	50 (58.1)	53 (61.6)	33 (64.7)	0.002*	0.151
No	109 (41.6)	114 (55.8)	36 (41.9)	33 (38.4)	18 (35.3)		
Respiratory transmission							
Yes	83 (27.9)	42 (16.3)	5 (5.8)	10 (11.6)	4 (7.8)	<0.001*	0.209
No	189 (72.1)	216 (83.7)	81 (84.2)	7 (88.4)	47 (92.2)		
Fecal‐oral route transmission							
Yes	29 (11.1)	22 (8.5)	10 (11.6)	10 (11.6)	2 (3.9)	0.465	0.069
No	233 (88.9)	236 (91.5)	76 (88.4)	76 (88.4)	49 (96.1)		
Kissing							
Yes	163 (62.2)	183 (70.9)	41 (47.7)	42 (48.8)	27 (52.9)	<0.001*	0.189
No	99 (37.8)	75 (29.1)	45 (52.3)	44 (51.2)	24 (47.1)		
**Transmission of respiratory infections**							
Air droplets							
Yes	239 (91.2)	237 (91.9)	78 (90.7)	78 (90.7)	49 (96.1)	0.811	0.046
No	23 (8.8)	21 (8.1)	8 (9.3)	8 (9.3)	2 (3.9)		
Fecal oral route							
Yes	48 (18.3)	33 (12.8)	5 (5.8)	4 (4.7)	1 (2.0)	<0.001*	0.172
No	214 (81.7)	225 (87.2)	81 (94.2)	82 (95.3)	50 (98)		
Respiratory secretions							
Yes	221 (84.14)	229 (88.8)	78 (90.7)	83 (96.5)	46 (90.2)	0.035*	0.118
No	41 (15.6)	29 (11.2)	8 (9.3)	3 (3.5)	5 (9.8)		
Sexual intercourse							
Yes	28 (10.7)	21 (8.1)	10 (11.6)	3 (3.5)	4 (7.8)	0.268	0.084
No	234 (89.3)	237 (91.9)	76 (88.4)	83 (96.5)	47 (92.2)		
Contact with contaminated hands							
Yes	115 (43.9)	56 (21.7)	10 (11.6)	20 (23.3)	13 (25.5)	<0.001*	0.258
No	147 (56.1)	202 (78.3)	76 (88.8)	66 (76.7)	38 (74.5)		
**Spread of diarrheal illnesses**							
Air droplets							
Yes	41 (15.6)	28 (10.9)	6 (7.0)	7 (8.1)	3 (5.9)	0.065	0.109
No	221 (84.4)	230 (89.1)	80 (93.0)	79 (91.9)	48 (94.1)		
Fecal oral route							
Yes	202 (77.1)	231 (89.5)	69 (80.2)	68 (79.1)	40 (78.2)	0.004*	0.144
No	60 (22.9)	27 (10.5)	17 (19.8)	18 (20.9)	11 (21.6)		
Respiratory secretions							
Yes	28 (10.7)	17 (6.6)	6 (7.0)	7 (8.1)	4 (7.4)	0.533	0.065
No	234 (89.3)	241 (93.1)	80 (93.0)	79 (91.9)	47 (92.2)		
Sexual intercourse							
Yes	39 (14.9)	29 (11.2)	11 (12.8)	10 (11.6)	2 (3.9)	0.259	0.084
No	223 (85.1)	229 (88.8)	75 (87.2)	76 (88.2)	49 (96.1)		
Contact with contaminated hands							
Yes	200 (76.3)	212 (82.2)	66 (76.7)	73 (84.9)	43 (84.3)	0.249	0.085
No	62 (23.7)	46 (17.8)	20 (23.3)	13 (15.1)	8 (15.7)		

* Statistically significant at *p* < 0.05.

### 3.2 Attitude of HCWs about HAI prevention

#### 3.2.1 Correlation of HCWs' professional nature and attitude scores

The professional attitude of HCWs toward the prevention of HAIs was significantly favorable in this study. Specifically, HCWs demonstrated a strong disagreement with the notion of considering catheters (*p* < 0.001) and surgical forceps (*p* < 0.001) as reusable equipment. Furthermore, HCWs strongly reported the importance of using gloves while in direct contact with patients (*p* < 0.001), and recognized the high risk of HAIs (*p* < 0.001). The study also found that HCWs had a considerably high favorable attitude (*p* < 0.001) toward the practice of hazard prevention to ensure that others were not put at risk (Table 2).

#### 3.2.2 Correlation of HCWs' experience and attitude scores

HCWs revealed a strong relationship between their level of experience and their attitudes toward the reuse of certain medical equipment. They strongly disagreed toward using catheters (*p* < 0.001), endoscopes (*p* < 0.001), surgical sponges (*p* < 0.024), and forceps (*p* < 0.003) as reusable equipment. Furthermore, HCWs recognized the importance of using goggles to prevent accidents, and their behaviors toward the prevention of hazards were favorable (*p* < 0.001). The study also found that HCWs recognized the high risk of HAIs (*p* < 0.001) and had a significantly favorable attitude (*p* < 0.001) toward preventing them (Table 6). About 72.13% of individuals had appropriate attitudes (Table 3).

**Table 6:** Correlation of healthcare workers (HCWs) practice and profession.

Practice	Profession (%)									
	Doctors	Nurses	Medical practitioners	Technicians	Pharmacists	Midwives	Allied health professionals	Other hospital staff	*p*‐Value	Cramer's *v*
**Wearing masks**										
All the time	326 (67.6)	6 (12.2)	22 (35.5)	6 (12.5)	21 (34.4)	0 (0)	8 (30.8)	9 (64.3)	<0.001*	0.304
Wear mask at suspected places	148 (30.7)	41 (83.7)	39 (62.9)	41 (85.4)	36 (59.0)	1 (100)	16 (61.5)	4 (28.6)		
Never	8 (1.7)	2 (4.1)	1 (1.6)	1 (2.1)	4 (6.6)	0 (0)	2 (7.7)	1 (7.1)		
**Using single needle for two pricks on a single patient**										
Yes	82 (17)	3 (6.1)	9 (14.5)	3 (6.3)	7 (11.5)	0 (0)	1 (3.8)	3 (21.4)	0.123	0.124
No	400 (83.0)	46 (93.9)	53 (85.5)	45 (93.8)	54 (88.5)	1 (100)	25 (96.2)	11 (78.6)		
**Using same medical equipment for another patient after sterilization**										
Yes	351 (72.8)	37 (75.5)	39 (62.9)	35 (72.9)	42 (68.9)	0 (0)	19 (73.1)	8 (57.1)	0.403	0.099
No	131 (27.2)	12 (24.5)	23 (37.1)	13 (27.1)	19 (31.1)	1 (100)	7 (26.9)	6 (42.9)		
**Immediate action during blood exposure accident**										
Don't know	50 (10.4)	3 (6.1)	7 (11.3)	1 (2.1)	11 (18.0)	0 (0)	4 (15.4)	4 (28.6)	0.153	0.114
Protect wound with bandage	399 (82.8)	39 (79.6)	50 (80.6)	43 (89.6)	43 (70.5)	1 (100)	20 (76.9)	10 (71.4)		
Rinse the eye thoroughly with water	33 (6.8)	7 (14.3)	5 (8.1)	4 (8.3)	7 (11.5)	0 (0)	2 (7.7)	0 (0)		
**Frequency of washing hands**										
Before and after touching a patient										
Yes	366 (75.9)	46 (93.9)	51 (82.3)	45 (93.8)	53 (86.9)	1 (100)	24 (92.3)	12 (85.7)	0.003*	0.171
No	116 (24.1)	3 (6.1)	11 (17.7)	3 (6.3)	8 (13.1)	0 (0)	2 (7.7)	2 (14.3)		
Before and after aseptic procedures										
Yes	332 (68.9)	46 (93.9)	41 (66.1)	44 (91.7)	37 (60.7)	0 (0)	24 (92.3)	13 (92.9)	<0.001*	0.230
No	150 (31.1)	3 (6.1)	21 (33.9)	4 (8.3)	24 (39.3)	1 (100)	2 (7.7)	1 (7.1)		
Only before and after having a meal										
Yes	50 (10.4)	1 (2.0)	3 (4.8)	2 (4.2)	4 (6.6)	0 (0)	0 (0)	1 (7.1)	0.183	0.117
No	432 (89.6)	48 (98)	59 (95.2)	46 (95.8)	57 (93.4)	1 (100)	26 (100)	13 (92.9)		
After body fluid exposure										
Yes	347 (72.0)	45 (91.8)	45 (72.6)	45 (93.8)	41 (67.2)	1 (100)	23 (88.5)	12 (85.7)	0.001*	0.182
No	135 (28)	4 (8.2)	17 (27.4)	3 (6.3)	20 (32.8)	0 (0)	3 (11.5)	2 (14.3)		
After touching a patient's immediate surroundings										
Yes	285 (59.1)	43 (87.8)	42 (67.7)	43 (89.6)	46 (75.4)	0 (0)	23 (88.5)	11 (78.6)	<0.001*	0.243
No	197 (40.9)	6 (12.2)	20 (32.3)	5 (10.4)	15 (24.6)	1 (100)	3 (21.4)	3 (21.4)		

* Statistically significant at *p* < 0.05.

### 3.3 Practice of HCW in HAI prevention

#### 3.3.1 Correlation of HCWs' professional nature and practice scores

A strong correlation was observed between handwashing and direct patient contact, both before and after (p 0.003), pre‐ and postaseptic procedures (*p* ≤ 0.001, *V* = 0.230), contact with immediate patient surroundings (*p* ≤ 0.001, *V* = 0.243), and exposure to body fluids (*p* < 0.001) (Table 6).

#### 3.3.2 Correlation of HCWs' experience and practice scores

Based on their experience, HCWs manifested a strong association between the practice of standard precautions and adherence to proper mask usage (*p* < 0.001) and regular handwashing before and after aseptic procedures (*p* < 0.001), direct patient contact (*p* < 0.001), exposure to body fluids (*p* < 0.001), and contact with patient surroundings (*p* < 0.001). Furthermore, avoiding the use of a single needle for more than one prick on a single patient (*p* < 0.001), usage of sterilized medical equipment (*p* < 0.001), and risk prevention during blood exposure accidents were also significantly practiced among HCWs (*p* < 0.001) (Table 7).

**Table 7:** Correlation of healthcare workers (HCWs) practice and experience.

Practice	Years of experience (%)						
	<1	1–5	5–10	10–20	>20	*p*‐Value	Cramer's *v*
**Wearing masks**							
All the time	158 (60.3)	178 (69.0)	20 (23.3)	24 (27.9)	18 (35.3)	<0.001*	0.249
Wear mask at suspected places	98 (37.4)	74 (28.7)	63 (73.3)	59 (68.6)	32 (62.7)		
Never	6 (2.3)	6 (2.3)	3 (3.5)	(3.5)	1 (2.0)		
**Using single needle for two pricks on a single patient**							
Yes	57 (21.8)	28 (10.9)	12 (14.0)	8 (9.3)	3 (5.9)	0.001*	0.159
No	205 (78.2)	230 (89.1)	74 (86)	78 (90.7)	48 (94.1)		
**Using same medical equipment for another patient after sterilization**							
Yes	161 (61.5)	205 (79.5)	64 (74.4)	64 (74.4)	37 (72.5)	<0.001*	0.171
No	101 (38.5)	53 (20.5)	22 (25.6)	22 (25.6)	14 (27.5)		
**Immediate action during blood exposure accident**							
Don't know	36 (13.7)	28 (10.9)	6 (7.0)	6 (7.0)	4 (7.8)	0.001*	0.137
Clean disinfect and protect the wound with bandage	205 (78.2)	223 (86.4)	71 (82.6)	69 (80.2)	37 (72.5)		
Rinse the eye thoroughly with water	21 (8.0)	7 (2.7)	9 (10.5)	11 (12.8)	10 (19.6)		
**Frequency of washing hands**							
Before and after touching a patient							
Yes	212 (80.9)	188 (72.9)	76 (88.4)	77 (88.4)	45 (88.2)	0.001*	0.162
No	50 (19.1)	70 (27.1)	10 (11.6)	9 (10.5)	6 (11.8)		
Before and after aseptic procedures							
Yes	150 (57.3)	196 (76.0)	71 (82.6)	74 (86.0)	46 (90.2)	<0.001*	0.265
No	112 (42.7)	62 (24.0)	15 (17.4)	12 (14.0)	5 (9.8)		
Only before and after having a meal							
Yes	36 (13.7)	12 (4.7)	4 (4.7)	7 (8.1)	2 (3.9)	0.001*	0.154
No	226 (86.3)	246 (95.3)	82 (95.3)	79 (91.9)	49 (96.1)		
After body fluid exposure							
Yes	169 (64.5)	202 (78.3)	70 (81.4)	72 (83.7)	46 (90.2)	<0.001*	0.197
No	93 (35.5)	56 (21.7)	16 (18.6)	14 (16.3)	5 (9.8)		
After touching a patient's immediate surroundings							
Yes	155 (59.2)	157 (60.9)	71 (82.6)	70 (81.4)	40 (78.4)	<0.001*	0.207
No	107 (40.8)	101 (39.1)	15 (17.4)	16 (18.6)	11 (21.6)		

* Statistically significant at *p* < 0.05.

### 3.4 HCWs practice and infection transmission risk

The binary logistic regression (BLR) model was found to be statistically insignificant based on the Hosmer and Lemeshow test, with a *p* > 0.05. The knowledge of transmission routes, coupled with the practice of reusing a single needle for multiple pricks, showed an odds ratio (OR) of 2.212 (95% CI: 0.956–5.119; *p* = 0.064). Regarding blood exposure accidents, those who did not know what to do in such situations had an OR of 1.082 (95% CI: 0.231–5.070; *p* = 0.921), while those who practiced protecting their wounds to prevent infection transmission showed an OR of 1.017 (95% CI: 0.269–3.843; *p* = 0.980). Those who frequently washed their hands to prevent infection transmission showed an OR of 1.02 (95% CI: 0.386–2.697; *p* = 0.967) (Table 8).

**Table 8:** Risk of infection transmission with reference to the healthcare workers' (HCWs) practice.

Practice	SE.	Sig.	Exp (B)	95% CI for Exp (B)	
				Lower	Upper
Wearing mask**s**					
All the time	0.664	0.001	0.109	0.03	0.401
Wear mask at suspected places	0.641	0.058	0.296	0.084	1.042
Using single needle for two pricks on a single patient					
Yes	0.428	0.064	2.212	0.956	5.119
Using same medical equipment for another patient after sterilization					
Yes	0.419	0.949	0.973	0.428	2.213
Immediate action during blood exposure accident					
Don't know	0.788	0.921	1.082	0.231	5.07
Protect wound with a bandage	0.678	0.98	1.017	0.269	3.843
Frequency of washing hands					
Before and after touching a patient	0.496	0.967	1.021	0.386	2.697
Before and after aseptic procedures	0.499	0.394	0.653	0.246	1.739
Only before and after having a meal	0.685	0.586	0.689	0.18	2.636
After body fluid exposure	0.521	0.028	0.318	0.114	0.883
After touching a patient's immediate surroundings	0.482	0.868	0.923	0.359	2.373

Abbreviations: CI, confidence interval; SE, standard error.

## 4 DISCUSSION

Infection control is a critical issue in healthcare facilities, with HCWs being the primary transmitters of infections. Therefore, this study aimed to assess the KAP of HCWs toward nosocomial infection prevention. The doctors (77.9%), medical practitioners (80%), and other hospital staff (77.2%) exhibited high knowledge scores compare to their attitude and practice toward the prevention of HAI. Kareem et al. also reported similar findings with more than half the number of doctors having less than 50% practice in evidence‐based medicine (EBM). Commonly reported impediments included the attitude of colleagues, inadequate skills, insufficient time, high flow of patients, and criticism fear.^20^ Our study revealed significantly higher practice scores among nurses (88.7%), technicians (75.1%), and allied health professionals (85.5%). In contrast, Maurya et al. reported knowledge scores of 11.9 in their study.^15^ The former study is a reflection of a single hospital, whereas the current results are representing the global nurses, and this could possibly indicate the disparities between the studies. Concerning EBM, Kaseka et al. identified moderate practice scores (57.8%) among nurses and midwives in a central hospital in Malawi.^21^ Clinical experience and the types of hospitals were the chief contributors to these scores.^15^ Regarding Allied Health professions and technicians, Mukhopadhyay et al reported that the HCWs' that were directly or indirectly involved in the laboratory diagnosis of COVID‐19 were more likely to exhibit good practices.^22^ These studies are evidence that the nature of profession and training plays a significant role in shaping the HCWs' practices.

Based on experience, the highest knowledge scores were spotted in highly experienced individuals (10–20 and >20 years). Among Nigerian HCWs, good knowledge was mainly observed in the 16–20 years experience category.^23^ We found a significant degree of good attitude amongst individuals having 1–5 years of experience. These results are inconsistent with hospital reports of Odisha, where higher attitude scores were recorded in ≥16 years of experience.^24^ The inconsistencies may be present due to the sample size distinction Concerning practice, we recorded peaked values in 10–20 years experienced HCWs, and lowest values in <1 years experienced HCWs. The overall KAP percentages were identified in HCWs with the highest experience. Indian researchers reflected a similar trend, where high practice scores were reflected in highly experienced HCWs.^25^

Correlational analysis manifested that a significant proportion of global HCWs exhibited high levels of knowledge concerning the transmission of microorganisms such as HCV, HDV, chickenpox, and shingles, as well as the dissemination of soft tissue infections, and direct contact spread of respiratory infections. Moreover, the respondents' understanding regarding the standard precautions of infection control was comparable to that reported in a study conducted by Engda, T in 2020, Ethiopia. Specifically, 59.5% of the respondents received training on nosocomial infections, while only 35.5% demonstrated good knowledge of nosocomial infections, and 40% exhibited good knowledge of the modes of transmission and risk factors for nosocomial infections.^26^ In another Ethiopian study, Bayleyegn et al. reported 90.2% of respondents with a high level of knowledge about preventing healthcare‐associated infections.^2^ Additionally, 96.6% of participants understood the imperative need to implement standard operating procedures (SOPs) to reduce HAI incidence. The statistics from Northwest Ethiopia manifested that 84.6% of respondents were knowledgeable about infection prevention.^27^ Moreover, 93.33% of participants demonstrated awareness of disinfectants and antiseptic preventive measures in connection with HAIs. Comparably, Pakistan's statistics accentuated 70% (*n* = 115) good knowledge of standard precautions for controlling nosocomial infections.^28^ The variation in the percentage of knowledge regarding the prevention of HAIs among HCWs can be influenced by the availability of educational programs and training, differences in the study population, and the priority placed on infection prevention by healthcare organizations.^2^

Findings from our study strongly indicated that HCWs possess a robust understanding of hand hygiene in infection prevention, and this knowledge tends to increase with years of experience. This finding is consistent with the study conducted in 2020 from northeast Ethiopia. Assefa et al. reported that 95.3% of the study participants had knowledge about the role of washing hands with soap or alcohol‐based antiseptics in reducing the transmission risk of nosocomial pathogens. In addition, HCWs with more than 5 years of work experience were 1.5 times more likely to have adequate knowledge compared to those with less experience. As HCWs gain experience, they become more insightful and proficient regarding infection control techniques. Thus, respondents with older age, longer work experience, and higher educational status tend to excel in infection prevention.^29^

Previous studies have reported substantial evidence of an association between professional work experience and knowledge regarding standard precautions. Specifically, as work experience increased, knowledge related to standard precautions also increased.^28^ Likewise, this study found that HCWs with greater experience were more likely to translate their knowledge into practice, as demonstrated by their adherence to infection prevention activities such as frequent hand washing before and after aseptic procedures, after touching patients' immediate surroundings, and wearing masks.

Importantly, our findings indicate that working experience is a significant factor associated with infection prevention practices. Specifically, HCWs with increased years of experience are three times more likely to engage in infection prevention activities than those with less experience. Assefa et al. in Northeast Ethiopia reported that 55% of healthcare providers had safe infection prevention practices.^29^ Agbana reported that only 53.4% of participants had received training on hand washing in the last 3 years of experience in Nigeria,^30^ while Gasabaet al. from Zimbabwe found that 79.3% of participants washed their hands and 75.9% were aware that the infection control manual guideline was available in their workplace.^5^ Our findings align with a study conducted in Uganda which reported that nurses' attitudes improved with greater work experience, making them better models for younger employees.^31^ Similarly, a study in Ethiopia found that HCWs with 10 or more years of experience were three times more likely to practice infection prevention and control than those with less than 5 years of experience. Furthermore, participants who had a positive attitude toward infection prevention and control practice were 10 times more likely to follow safe practices.^22^

Previous studies have reported mixed findings on infection prevention practices among HCWs. Some studies have shown good adherence to practices, such as wearing masks (83.7%) and hand hygiene (98.3%, 91.8%), while others found lower rates of compliance (45.5% in a study by SisayFogaSebro^32^). Additionally, a study from Bahir Dar, Ethiopia, reported 54.2% of healthcare providers practicing nosocomial infection prevention,^2^ and Gezie et al. in Northeast Ethiopia found less than one‐fourth of the study population (23%) demonstrating good practices toward HAI prevention.^6^ These findings provide valuable context but should be interpreted cautiously as they may differ in population, methodology, and settings compared to our study.

The positive attitude of HCWs is an essential requirement for preventing nosocomial infections and a crucial aspect in preventing cross‐infections. Our study's findings indicate that HCWs have a positive attitude toward their profession, as evidenced by their preference for using gloves (*p* < 0.001, Cramer's *v* = 0.197) during direct contact with patients, removing hazards immediately to protect others from risks, and their understanding that healthcare professionals are at high risk of nosocomial infections. Contrastingly, a study conducted in Nigeria by Babatola et al. identified only 56.7%^33^ of HCWs with a good attitude toward preventing nosocomial infections. Similarly, a study from Bahir Dar city in Ethiopia reported 55.6% of HCWs that exhibited a good attitude^28^ toward preventing such infections. Comparably, our findings are similar to a study conducted by Sai SubhakarDesu in India in 2023, which reported that 74.5% of participants had a positive attitude toward preventing nosocomial infections. Additionally, 89.2% of participants agreed that guidelines for preventing HAIs should be strictly followed, and hand hygiene measures should be taken to reduce the risk of infections after treating patients. A total of 80.8% of respondents often or always used gloves and practiced hand hygiene measures after removing gloves.^34^ Similarly, a study from Northeast Ethiopia by Jemal S in 2019 reported that 69.23% of HCWs had a positive attitude toward infection prevention, while 30.77% had a negative attitude. However, the majority of study participants, that is, 71.43%, did not consider that all HCWs and the community are at risk of infection.^35^ Engda et al. in Ethiopia revealed 36% of study participants with a good attitude toward preventing and controlling nosocomial infections.^26^ In contrast, Alhassan et al. from Ghana, in 2021, reported that 97.4% (*p* ≤ 0.001) of respondents agreed to wash their hands following the removal of gloves. About 95.5% of respondents believed that following prevention guidelines would reduce HAI rates, and 86% agreed that following the SOPs would decrease the risk of contamination.^36^

Furthermore, Unakal et al. conducted a study in three hospitals in Trinidad and Tobago in 2017, which indicated that 53.3% of participants had a positive attitude toward infection prevention and control. Additionally, 87.7% of HCWs agreed that a new pair of gloves should be worn for each new patient attended. This translated to a practice level of 56.0%, demonstrating the influence of attitude on practice.^37^ The variations in research results can be attributed to several factors, including the academic background of the participants, the sample size, and the availability and implementation of protocols for preventing HAIs. In some settings, hand hygiene may be viewed as a minor concern and may not be given sufficient attention, particularly in nonsurgical and noninvasive procedures.

Organizational support factors, such as the presence of an infection control team, supply of disinfectants, and visible information (leaflets and posters) about HAIs, play a significant role in shaping behavior. It puts HCWs into a reminder about the threats and the impact of HAIs in line with the health beliefs model, increasing the “willingness to act.” The majority of HCWs in our study wore gloves while having direct contact with patients, indicating a positive trend toward safe practices. In contrast, a study from Italy reported that only 57.3% of HCWs changed gloves to prevent the transmission of infection.^38^ The availability of gloves may reflect these inconsistencies, as improved availability allows more accessibility. Adherence to safe infection prevention practices is mandatory for both patients and HCWs, as it reduces the incidence of HAIs. However, a recent study reported unsatisfactory levels of good practice, indicating the need for continued efforts to improve infection prevention and control measures.^2^

It is important for hospital administrators to foster a culture that prioritizes adherence to recommended hand hygiene practices and infection prevention measures. This can be achieved by providing visible support and adequate resources for continuing education programs that are tailored to the specific needs of healthcare personnel. Despite the fact that most healthcare providers in our study had good knowledge and a favorable attitude toward HAI prevention, there is still room for improvement. Therefore, ongoing efforts are needed to ensure that infection prevention practices are consistently implemented in healthcare settings.

## 5 LIMITATIONS

There are certain limitations to our study, such as the use of a self‐administered survey questionnaire as it may introduce the potential for response bias (participants may not respond truthfully or accurately due to social desirability bias, where they provide socially acceptable answers rather than their true perceptions), and the exclusion of housekeeping staff who may also be a source of transmitting HAIs. Additionally, social desirability bias may have influenced the results to some extent. To address these limitations, large‐scale multicenter studies are needed. The predominance of doctors in the study sample may lead to an overrepresentation of their perspectives, and not knowing the specific departments where HCW work could limit the generalizability of findings to different healthcare settings. Additionally, cultural norms and beliefs shaping attitudes toward practices like mask‐wearing or glove use, resource availability affecting adherence to guidelines in resource‐limited settings, communication barriers hindering information dissemination, and organizational support may impact infection control programs' effectiveness, leading to disparities in training opportunities and cultural attitudes toward infection control, thereby affecting KAP levels. Understanding and addressing these factors are crucial for designing targeted strategies to enhance infection prevention practices among HCWs globally, ultimately reducing nosocomial infections.

## 6 SUGGESTIONS

To address the gaps identified in knowledge, attitudes, and practices related to infection prevention among HCWs, we propose the following educational approaches:

1. *Regular Training Programs*: Implement regular and comprehensive training programs on infection prevention for all HCWs, including doctors, nurses, technicians, and allied health professionals. These programs should be evidence‐based, interactive, and tailored to the specific needs of each professional group.
2. *Simulation and Role‐Modeling*: Utilize simulation‐based training and role‐modeling to demonstrate best practices in infection prevention. Practical demonstrations can be more effective in reinforcing the importance of adherence to guidelines.
3. *Infection Control Champions*: Designate infection control champions within each department or unit to act as leaders and advocates for infection prevention. These champions can lead by example, provide guidance, and promote a positive culture of infection control.
4. *Regular Feedback and Audits*: Conduct regular audits and provide timely feedback to HCWs on their infection prevention practices. Feedback can help identify areas for improvement and motivate HCWs to adhere to best practices.
5. *Multidisciplinary Training*: Foster a multidisciplinary approach to infection prevention training, where different healthcare professionals collaborate and learn from each other's experiences. This can enhance teamwork and promote a unified approach to infection control.

## 7 CONCLUSION

In summary, our study accentuated the impact of HCWs' knowledge regarding the mode of infection transmission on their infection prevention practices. The study provides valuable insight into the KAP scores of HCWs, based on years of experience and profession. The study findings indicate that HCWs globally, including doctors, medical practitioners, nurses, and other hospital staff, have a good understanding and positive attitude toward the prevention of HAIs or nosocomial infections. Moreover, their knowledge was observed to be reflected in their practices. However, it is recommended that educational seminars and awareness programs be conducted annually to ensure the retention of knowledge, attitudes, and practices among HCWs of different categories. These educational programs would help improve adherence to barrier protection measures such as hand washing, the use of gloves, and hand disinfection. It is also important for every institution to have proper guidelines for HCWs, and a regular system of monitoring infection rates and disseminating data to form a link between the management and the HCWs to improve strategies for HAI prevention.

## AUTHOR CONTRIBUTIONS

**Elham M. Khatrawi**: Conceptualization; data curation; formal analysis; investigation, Visualization; writing—original draft; writing—review and editing. **Priyadarshi Prajjwal**: Conceptualization; data curation; formal analysis; investigation; visualization; writing; original draft; writing—review and editing. **Muhammad Farhan**: Formal analysis; investigation; visualization; writing—original draft; writing—review and editing. **Pugazhendi Inban**: Formal analysis; investigation; visualization; writing—original draft; writing—review and editing. **Shraddha Gurha**: Visualization; writing—original draft; writing—review and editing. **Saud M. S. Al‐ezzi**: Writing—original draft; writing—review and editing. **Mohammed D. M. Marsool**: Writing—original draft; writing—review and editing. **Prerna Ahuja**: Writing—original draft; writing—review and editing. **Mohammed A. Mateen**: Writing—original draft; writing—review and editing. **Felix O. Aina**: Writing—original draft; writing—review and editing. **Omniat Amir**: Writing—original draft, writing—review and editing.

## CONFLICT OF INTEREST STATEMENT

The authors declare no conflict of interest.

## TRANSPARENCY STATEMENT

The lead author Elham Mohammed Khatrawi affirms that this manuscript is an honest, accurate, and transparent account of the study being reported; that no important aspects of the study have been omitted; and that any discrepancies from the study as planned (and, if relevant, registered) have been explained.

## Supporting information

Supporting information.

## Data Availability

The authors confirm that the data supporting the findings of this study are available within the article. Raw data that support the findings are available at Zenodo under title: Analyzing the knowledge, attitude, and practices of healthcare workers toward the awareness of nosocomial infections: A global survey. doi: 10.5281/zenodo.7845444.
