# Supplementary material for: Evaluating the knowledge, attitudes, and practices of healthcare workers regarding high‐risk nosocomial infections: A global cross‐sectional study
Source: Health Sci Rep. 2023 Sep 11;6(9):e1559. doi: 10.1002/hsr2.1559 (PMC10494663; doi:10.1002/hsr2.1559)
Supplement: Supplementary file 1 — Supporting information. [file HSR2-6-e1559-s001.pdf]

Survey Questionnaire for Healthcare Workers

You are in direct contact with patients on a daily basis and this is why we are interested in your opinion on health care-associated infections and

- It should take you about 3 to 4 minutes to complete this questionnaire.
- Each question is provided with multiple choice. Some questions may require one response, others may involve more than one response.

Please read the questions carefully and then respond spontaneously. Your answers are anonymous and will be kept confidential

This survey will help to analyze the transmission risk of frequently encountered nosocomial infections. This survey is being conducted for research purposes and requires only a few minutes of your time to complete with an electronic device. There are no risks to participating in the survey and your participation is completely voluntary. You may withdraw from the survey at any time. No identifiable information will be gathered and the information you provide will remain anonymous. Thank you for your time

CONSENT

☐ I have read and I understand the provided information. I understand that my participation is voluntary and that I am free to withdraw at any time, without giving a reason and without cost. I voluntarily agree to take part in this study

Country___________

City______________

1- Definition of 'Routes of Microbial Transmission'?
   a- Vehicles
   b- mode of infection spread
   c- Transfer from one country to another

2- What is your Age Group?
   a. 17- 25
   b. 25-35
   c. 35-45
   d. 45-60

3- What is your profession?

   a. Doctors
   b. Nurses
   c. Medical practitioners
   d. Technicians
   e. Pharmacists

f. Midwives
g. allied health professionals
h. Other hospital staff

4- Your years of Experience in Healthcare profession

a. <1
b. 1-5
c. 5-10
d. 10-20
e. >20

Knowledge Based

5- What of the following microbes that is mainly transmitted through blood? (Check all that apply)

a. Influenza
b. Hepatitis C and D Virus
c. Tuberculosis causing bacterium
d. Chicken pox and Shingles (caused by Herpes Virus)
e. HIV

6- Can infection causing germs be killed by 70% alcohol?

a- Yes
b- No

7- Can a person who looks healthy is capable to transmit any infection
a- Yes
b- No

8- Can sharing food items with patients spread viral or bacterial infections
a- Yes
b- No

9- How to clean hands while caring for patients with vomiting or diarrheal illnesses?
a- Alcohol
b- Soap
c- Any of them

10- Can we bend, shear, break, or recap disposable needles or remove from disposable syringes?
a- Yes
b- No

11- Can we empty the contents of the sharp's disposal container into another container?
a- Yes
b- No

12- Do only the elderly, comorbid patients and people with weak immune system are affected by hospital acquired illnesses?
   a- Yes
   b- No

13- Medical Gowns should be worn during surgical procedures only
   a- True
   b- False

14- How skin and soft tissue infections can be transmitted (Check all that apply)
   a- Skin contact
   b- Sexual intercourse
   c- Respiratory Transmission
   d- Fecal-oral Route transmission
   e- Kissing

15- How respiratory infections can be transmitted (Check all that apply)
   a- Air Droplets
   b- Fecal oral route
   c- Respiratory secretions
   d- Sexual intercourse
   e- Contact with contaminated hands

16- How diarrheal illnesses can be spread? (Check all that apply)
   f- Air Droplets
   g- Fecal oral route
   h- Respiratory secretions
   i- Sexual intercourse
   j- Contaminated hands

Practice Based

17- How frequent you wash your hands (check all that apply)
   a- Before and after touching a patient
   b- before and after aseptic procedures
   c- Only before and after having a meal
   d- after body fluid exposure
   e- after touching a patient’s immediate surroundings

18- When you should wear Mask
   a- At all times in hospital
   b- At Suspected places
   c- Never

19- Do you use a single needle for two pricks on a single patient?

  a- Yes
  b- No

20- Will you use a same medical equipment for another patient after sterilization?
a- Yes
b- No

21- What you will do for a blood exposure accident?
  a- Do not Know
  b- Clean, disinfect and protect the wound with the bandage
  c- Rinse the eye thoroughly with water

Attitude Based

22- In your opinion, what is the average percentage of healthcare workers in your hospital who will develop a health care-associated infection (between 0 and 100%)?

a. 0-25%
b. 25-50%
c. 50-75%
d. 75-100%
e. I Do not know

23- In your opinion, what are the reusable equipment in this list? (Check all that apply)
a- Syringes
b- Stethoscope
c- Catheters
d- Endoscope
e- Surgical Sponges
f- Surgical Forceps

24- We can wear similar gloves while contacting different patients
  a- True
  b- False

25- Will you prefer wearing gloves while having a direct contact when a patient seems healthy?
  a- Yes
  b- No

26- In your perception, what is the best way of dealing with a hazard to ensure others are not put at risk?
  a) Remove it immediately
  b) Leave it for others to sort out

c) Place a barrier tape around it
d) Display a notice or warning sign

27- Should googles be worn while cutting catheter bags, cleaning bedpans and emptying suction cups?

a- Yes
b- No
